# Association between post-extubation upper airway obstruction symptoms and airway size measured by computed tomography: a single-center observational study

**DOI:** 10.1186/s12873-022-00615-7

**Published:** 2022-03-31

**Authors:** Mafumi Shinohara, Masayuki Iwashita, Takeru Abe, Ichiro Takeuchi

**Affiliations:** Advanced Critical Care and Emergency Center , Yokohama City University Medical Center, 4-57 Urafunecho Minamiku, Yokohama, Kanagawa 232-0024 Japan

**Keywords:** Intubation, Airway extubation, Airway management, Tracheal stenosis, Airway obstruction, Post-extubation stridor

## Abstract

**Background:**

Computed tomography (CT) is often performed to assess patients; however, little is known about how airway size measured by CT scan imaging might influence the occurrence of post-extubation upper airway obstruction.

**Methods:**

This study aimed to evaluate the association between airway size measured by CT and the incidence of post-extubation upper airway obstruction symptoms for each sex. This single-center observational study was conducted at a tertiary emergency medical center/severe trauma center with a 12-bed intensive care unit. We enrolled consecutive adult patients (aged ≥ 20 years), who were intubated in the emergency room, between January 2016 and March 2019. Patients who underwent a CT scan of the glottic region within three hours before and after intubation were included in the analysis. For each sex, we first divided the patients into two groups: those who had post-extubation stridor, hoarseness, or both and those who had no such symptoms. Then, we compared the two groups using the Mann–Whitney U test and Fisher’s exact test. Univariate and multivariate logistic regression analyses were also performed.

**Results:**

During the 39 months, 855 patients were enrolled in this study. A total of 217 patients underwent CT of the glottic region within three hours before and after intubation. Five patients had no records of symptoms after extubation. Thus, we analyzed data from 212 patients. This study included 144 males and 68 females. In female patients, the median [inter-quartile range] (average) of the transverse diameter of the glottis/endotracheal tube outer diameter (OD) ratio was smaller in patients with post-extubation upper airway obstruction symptoms than in patients without the symptoms (1.00 [1.00–1.00] (0.9572) vs. 1.00 [1.00–1.00] (1.00296), respectively; *p* = .013). Multivariate logistic regression analysis showed that the glottis/tube OD ratio < 1 was associated with the symptoms in females (odds ratio: 95% confidence interval, 5.68: 1.04–30.97). There was no relation between the airway sizes and the symptoms in male patients.

**Conclusions:**

In female patients, no gap between the endotracheal tube and the vocal codes or the glottic transverse diameter being smaller than the endotracheal tube OD on CT scan was associated with post-extubation upper airway obstruction symptoms.

## Background

Preventing post-extubation upper airway obstruction is clinically important because it can cause reintubation and subsequently increase patient mortality and morbidity [1–4]. Stridor and hoarseness are considered clinical manifestations of upper airway obstruction symptoms after extubation. The incidence of post-extubation stridor and hoarseness has been reported to range from 1.5% to 26.3% [5]. In emergency settings, the risk of post-extubation upper airway symptoms is higher and is seen in 29%–31% of patients [6, 7].

Female sex, prolonged intubation, and an increased number of intubation attempts were risk factors for post-extubation stridor and laryngeal edema [4–9]. In particular, female sex is a well-known risk factor for post-extubation stridor [6, 8, 10–13]. Airway size is a major risk factor in females because most of them have anatomically smaller airways than males [14, 15]. Using endotracheal tubes (≥ 7.0 mm) in females was a risk factor for post-extubation upper airway obstruction [16–18]. Several other factors are involved in post-extubation upper airway obstruction, including mechanical stimulation of the tracheal tube on vocal codes and around the glottis mucous membrane, vocal cord paralysis, increased secretions, and deterioration of laryngeal function. On the other hand, image evaluation such as computed tomography (CT) or ultrasound are usually used to measure airway size. In addition, laryngeal ultrasonography has been studied as an evaluation method for post-extubation stridor [9, 19]. Generally, only a trained physician or radiologist can perform laryngeal ultrasonography. In emergency settings, CT scans are often performed to assess patients, but little is known about how sex and airway size measured by CT scan imaging might interact with the occurrence of post-extubation upper airway obstruction. We hypothesized that a small airway size measured by CT might be associated with post-extubation upper airway obstruction symptoms.

## Methods

### Setting

The aim of this study was to evaluate the association between the airway size measured by CT and the incidence of post-extubation upper airway obstruction symptoms for each sex. We conducted an observational single-center study. We accumulated cases prospectively and analyzed the data retrospectively.

Our hospital is a tertiary emergency medical/severe trauma center with a 12-bed mixed intensive care unit (ICU) located in Yokohama, Japan; a standard urban emergency center. We had 27 full-time physicians (14 board-certified acute care physicians and 9 board-certified critical care physicians) at the start of this study. The average numbers of annual ambulances and patients who received mechanical ventilation in our emergency center were 1261 and 536 per year, respectively.

### Patients

We enrolled consecutive adult patients (aged ≥ 20 years), who were intubated in the emergency room by an emergency physician or a resident supervised by an emergency physician, from January 2016 to March 2019. Patients who underwent CT scan of the glottic region within three hours before and after intubation were included for analysis. Patients who underwent tracheostomy, were transferred, or died before the first attempt to extubate were excluded. We used oral tracheal tubes with a subglottic drainage lumen (Taper Guard Evac; Medtronic, Minneapolis, MN, USA) or standard oral tracheal tubes with a stylet (Taper Guard with stylet; Medtronic) depending on device availability.

### Study procedures

We accumulated the cases prospectively in chronological order and clerks who were not involved in this study distributed the paper database form for all eligible patients to avoid selection bias. An attending physician or a physician in charge recorded the following characteristics at the time of intubation: age, sex, height, body weight, reason for intubation, endotracheal tube size and type, history of tracheostomy and/or prolonged (> 2 weeks) intubation, number of intubation attempts, the intubation doctor’s years of experience (junior resident: 1–2 years, senior resident: 3–5 years, 6 years and more), use of a sedative drugs and neuromuscular blocking agents at intubation. The size of endotracheal tube used, as well as the use of sedative drugs and neuromuscular blocking agents at intubation were decided by an attending physician based on the patient’s condition. The ICU nurses checked the endotracheal cuff at least once every 8 h, and cuff pressure was maintained at 20–24 cm H_2_O. The timing of extubation was decided by attending physicians. The doctor who performed the extubation assessed and recorded whether the patient had stridor and hoarseness, or both after extubation. To minimize the observer bias, the post-extubation upper airway symptoms was confirmed by multiple doctors including those other than researchers of this study, as much as possible. The doctor who performed the extubation also recorded the use of steroids and the presence of a cuff leak before extubation.

Stridor was defined as a high-pitched inspiratory wheeze with respiratory distress. Hoarseness was defined as changes in voice quality and difficulty in speaking with respiratory distress, regardless of whether medical intervention was required. Cuff-leak test was performed as a qualitative test defined as an audible leak while the endotracheal balloon was deflated.

To minimize observer bias, the transverse diameters of the glottis and cricoid cartilages were measured using CT images by authors who were blinded to post-extubation symptoms. Position of the vocal cords was identified as the area where the thyroid cartilage and vocal cords were visible in the images. The transverse diameters of the glottis were measured as the distance between the vocal cords at widest point in the image. When there were multiple images in which the thyroid cartilage and vocal cords were visible, we selected the one with the narrowest diameter measured. When there was no gap between the endotracheal tube and the vocal cords in the patients that had been already intubated at the time of CT scan, the transverse diameters of the glottis were regarded as same with the outer diameter of the tracheal tube. The value obtained by dividing the transverse diameter of the glottis by the outer diameter (OD) of the endotracheal tube was used as the index of the endotracheal tube size by the airway size. Our primary outcome was post-extubation stridor and hoarseness, or both. At the time of discharge, an attending physician recorded that unplanned reintubation within 48 h, and hospital mortality.

### Statistical analysis

We compared the post-extubation upper airway obstruction symptoms in males and females using a chi-squared test. Then, we performed further analyses on the males and females separately, because airway size differed depending on sex. For each sex, we first divided the patients into two groups: those who had post-extubation stridor and/or hoarseness and those who had no such symptoms. The quantitative variables were expressed as the median [inter-quartile range: IQR] and compared using the Mann–Whitney U test. For the categorical variables, the comparisons were performed using the Fisher’s exact test. Univariate logistic regression analysis was used to evaluate the risk of post-extubation upper airway obstruction symptoms. For logistic regression analysis, we used the transverse diameter of glottis/ endotracheal tube OD ratio < 1 as the variable representative of the ratio of airway size to tube size, because the physical contact of endotracheal tube to tracheal membrane is the most likely mechanism of post-extubation airway edema, and presence of a gap between endotracheal tube and the vocal cords was the most important. A multivariable logistic regression model was applied using intubation attempts, duration of intubation, and the transverse diameter of glottis/tube OD ratio < 1. Regarding the independent variables in multivariable logistic regression analysis, we selected one of the most significant variables from the three areas, such as intubation procedure, patient condition during intubation and airway size, to avoid multicollinearity. We defined multiple intubation attempts as three and more attempts [20, 21]. We performed the multivariable logistic models’ goodness of fit and discrimination ability using the Hosmer–Lemeshow test and the c statistic. We excluded patients with missing data from the analysis. Statistical significance was set at *p* < 0.05. All statistical analyses were performed using STATA software (Stata/SE 13.0, StataCorp LLC, TX, USA).

This study was approved by an Institutional Review Board, the Ethics Committee of the Yokohama City University Medical Center (D1506007, approval date 17th July 2015). Requirement of informed consent from the patients was waived by the Ethics Committee of the Yokohama City University Medical Center /IRB because of the observational study design.

## Results

**Fig. 1:**
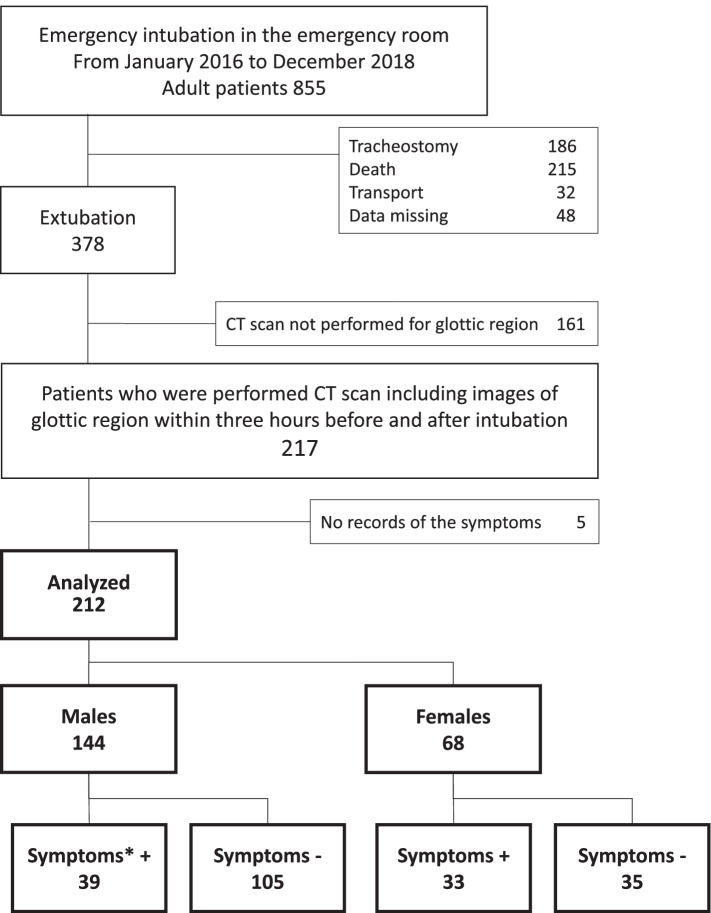
Patients flow diagram. Symptoms included stridor and/or hoarseness

During the period of the study, 855 patients were enrolled. The patient flow diagram is shown in Fig. 1. There were 217 patients who were performed CT scan of the glottic region within three hours before and after intubation. There were five patients whose records of post-extubation upper airway obstruction symptoms were lost. Thus, we analyzed data from 212 patients. There were 144 males and 68 females. The incidence of post-extubation upper airway obstruction symptoms was significantly different between females (49%) and males (27%) (*p* = .002). Patient characteristics according to sex are shown in Table 1. Trauma was the most common reason for intubation (*n* =108, 51%). We used sedative drugs in 173 patients (82%). Tracheal tube-type data were missing for three patients, and all the remaining 209 patients were intubated with oral tracheal tubes with a subglottic drainage lumen. In males, 98 (68%) patients were intubated with 7.5 mm endotracheal tubes. In females, 46 (68%) patients were intubated with 7.0 mm endotracheal tubes. The number of patients who were intubated on the first or second attempts and those intubated on the third or later attempts were 203 (96%) and 7 (3%), respectively. A total of 72 patients (34%) presented with stridor and/or hoarseness after extubation, and 140 patients did not have symptoms of upper airway obstruction after extubation. Seven patients (3%) required unplanned reintubation within 48 h. Among the seven patients, three patients who were reintubated because of upper airway obstruction had hoarseness immediately after extubation. In addition, four other patients required unplanned reintubation due to respiratory failure or deterioration of consciousness, and not due to upper airway obstruction.

**Fig. 2:**
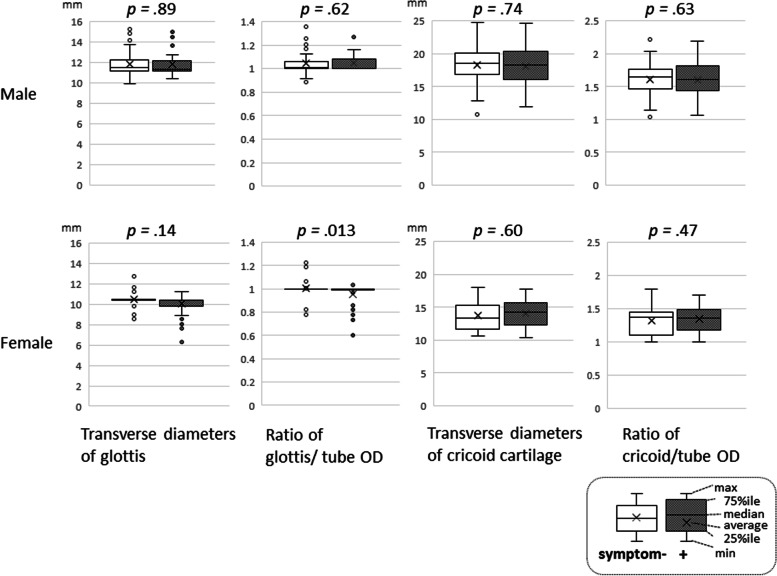
The tracheal sizes with or without symptoms

Tracheal sizes by sex, with or without symptoms, are shown in Fig. 2. The median [IQR] ratio of the transverse diameter of the glottis was 11.36 [11.20-12.15] vs. 11.49 [11.20-12.28] mm in males and 10.40 [9.80-10.40] vs. 10.40 [10.40-10.45] mm in females, with and without the symptoms, respectively. In male patients, there were no changes in the airway sizes of patients with or without symptoms. In female patients, the median [IQR] (average) of the transverse diameter of the glottis/tube OD was smaller in patients with symptoms than in patients without symptoms (1.00 [1.00-1.00] (0.9572) vs. 1.00 [1.00-1.00] (1.00296), respectively; *p* = .013).

We performed univariate logistic regression analysis and used post-extubation upper airway obstruction symptoms as objective variables (see Table 2).

There were no factors associated with upper airway obstruction symptoms after extubation in univariate logistic regression analysis.

Multivariate logistic regression analysis showed that the ratio of the glottis/tube OD < 1 was associated with postextubation upper airway obstruction symptoms in females (odds ratio [OR]: 95% confidence interval [CI], 5.68: 1.04-30.97) (Table 3).

**Table 1:** Comparison of patients’ characteristics with and without postextubation upper airway obstruction symptoms by sex

Characteristics	Males (*n* =144)		*p* value	Females (*n* =68)		*p* value
	Numbers (%) or median (IQR)			Numbers (%) or median (IQR)		
	Symptoms + (*n*=39)	Symptoms - (*n*=105)		Symptoms + (*n*=33)	Symptoms - (*n*=35)	
Age, years	55 (37-75)	55 (40-69)	.97	53 (39-73)	56.5 (40.25-80.25)	.55
Height, cm	170 (163-174)	170 (165-175)	.40	156 (150-161)	155 (146.5-158)	.16
Reasons of intubation			.32			.90
Trauma	18 (46%)	57 (54%)		17 (52%)	16 (46%)	
Deterioration of consciousness	9 (23%)	24 (23%)		9 (27%)	12 (34%)	
Pneumonia	0 (0%)	4 (4%)		2 (6%)	1 (3%)	
Sepsis	1 (3%)	5 (5%)		1 (3%)	2 (6%)	
Cardiac arrest	5 (13%)	4 (4%)		3 (9%)	2 (6%)	
Others	6 (15%)	11 (11%)		1 (3%)	2 (6%)	
Tube size (inner diameter)			.75			1.00
6.0 mm	0 (0%)	0 (0%)		0 (0%)	1 (3%)	
6.5 mm	0 (0%)	1 (1%)		4 (12%)	4 (11%)	
7.0 mm	2 (5%)	2 (2%)		23 (36%)	23 (66%)	
7.5 mm	27 (69%)	71* (68%)		6 (18%)	7 (20%)	
8.0 mm	10* (26%)	39 (37%)		0 (0%)	0 (0%)	
8.5 mm	0 (0%)	1 (1%)		0 (0%)	0 (0%)	
History of tracheostomy or prolonged intubation	0 (0%)	0 (0%)	-	0 (0%)	1 (3%)	1.00
Number of intubation			.053			.45
1	31 (79%)	95 (91%)		25 (76%)	30 (86%)	
2	7 (18%)	5 (4%)		5 (15%)	5 (14%)	
≥ 3	1 (3%)	4 (4%)		2 (6%)	0 (%)	
The intubation doctor’s years of experience			.11			.61
Junior resident: 1-2 years	1 (3%)	3 (3%)		2 (6%)	0 (0%)	
Senior resident: 3-5 years	5 (13%)	29 (28%)		7 (21%)	7 (20%)	
Senior doctor: 6 years and more	32 (82%)	66 (63%)		24 (73%	22 (63%)	
Unknown	1 (3%)	7 (7%)		0 (0%)	6 (17%)	
Sedative drugs use at intubation	33 (85%)	89 (85%)	1.00	25 (76%)	26 (74%)	1.00
Neuromuscular blocking agents use at intubation	32 (82%)	82 (78%)	.82	29 (88%)	28 (80%)	.51
Steroids use before extubation	2 (5%)	4 (4%)	.66	6 (18%)	5 (14%)	.75
Absence of cuff-leak before extubation	0 (0%)	2 (2%)	1.00	1 (3%)	0 (0%)	.48
Duration of Intubation, days	4 (3-7)	3 (2-6)	.10	3 (2-9)	4 (2-6)	.81
Unplanned reintubation within 48 hours	1 (3%)	4 (4%)	1.00	2 (6%)	0 (0%)	.23
Hospital mortality	0 (0%)	2 (2%)	1.00	0 (0%)	1 (3%)	1.00

**Table 2:** Univariate logistic regression analysis for postextubation upper airway obstruction symptoms

Factors	**Male**			**Female**		
	OR^a^	[95% CI^b^]	*p* value	OR^a^	[95% CI^b^]	*p* value
Intubation attempts ≥ 3 times	2.26	[0.57–8.91]	.24		Not calculated	
Duration of Intubation	1.09	[0.96–1.24]	.17	1.08	[0.93–1.25]	.31
Transverse diameters of glottis	1.06	[0.73–1.54]	.76	0.59	[0.34–1.06]	.076
Transverse diameters of 5 mm under glottis	1.09	[0.86–1.38]	.46	0.90	[0.50–1.59]	.71
Transverse diameters of cricoid cartilage	0.98	[0.84–1.14]	.80	1.08	[0.85–1.37]	.52
Ratio of glottis/ tube OD^c^ < 1		Not calculated		4.44	[0.85–23.21]	.077

^a^*OR* Odds ratio, ^b^*CI* Confidence interval, ^c^*OD* Outer diameter

**Table 3:** Multivariate logistic regression analysis for postextubation upper airway obstruction symptoms

Factors	**Male**			**Female**		
	OR^a^	[95% CI^b^]	*p* value	OR^a^	[95% CI^b^]	*p* value
Intubation attempts ≥ 3 times	2.81	[0.66- 11.96]	.16		Omitted	
Duration of Intubation	1.09	[0.96- 1.24]	.19	1.11	[0.94–1.30]	.22
Ratio of glottis/ tube OD^c^ < 1		Omitted	.93	5.68	[1.04–30.97]	.045
Hosmer–Lemeshow goodness of fit test			0.11			0.22
c statistics [95% CI]	0.61	[0.50–0.72]		0.61	[0.46–0.75]	

^a^*OR* Odds ratio, ^b^*CI* Confidence interval, ^c^*OD* Outer diameter

## Discussion

In this study, we revealed that no gap between endotracheal tube to vocal codes and the glottic transverse diameter is smaller than the endotracheal tube outer diameter in CT scan was the risk factor of post-extubation upper airway obstruction symptoms in females. In contrast, airway size was not associated with post-extubation stridor in males. The findings suggest that caution should be exercised when choosing a tube size during intubation depending on the patient’s sex.

Previous studies have reported that female sex, prolonged intubation, and an increased number of intubation attempts were risk factors for post-extubation stridor and laryngeal edema [4–9]. In addition, using endotracheal tubes (≥ 7.0 mm) in female patients was reported to be a risk factor for post-extubation upper airway obstruction in postoperative patients [16–18]. Our results add a new finding that measurement of airway size in CT scan may become predict the risk for developing post-extubation upper airway obstruction symptoms.

Reasons which explain the greater risk of post-extubation laryngeal edema seen in females has been discussed [6, 8, 10–13]. Airway size is one of the biggest reasons, since anatomically, most females have smaller airways than males [14, 15]. In this study, we mainly used 7.0 mm endotracheal tubes with subglottic drainage lumens for females. The endotracheal tubes with and without subglottic drainage lumens have different outer diameters even if they have the same inner diameter. In the products, the outer diameter of the 7 mm tracheal tubes with subglottic drainage lumen was 10.4 mm. In our results, the median [IQR] transverse diameters of the glottis were 10.4 [9.9–10.4] mm in females. Then, 7 mm tracheal tubes with subglottic drainage lumens may be too large for females.

There were some reports that a 7 mm tracheal tube was large for females basing from reports of symptoms of post-extubation upper airway discomfort, such as hoarseness or stridor [15–17]. However, other factors must be considered when selecting the tube size, respiratory condition, amount and nature of secretions, and use of bronchoscopy. Small endotracheal tubes are often insufficient for suctioning highly mucinous secretions or when scanning using bronchoscopy. In addition, we select the tubes with subglottic drainage lumens from the aspect of decreasing ventilator associated pneumonias. Our findings are useful for identifying patients at high risk of developing post-extubation upper airway obstruction and in endotracheal tube selection.

Our study had several limitations. First, this was a single-center study with a limited sample size. In addition, all included patients were Asian. Airway size is known to be associated with patient height [14]. It is unclear whether our results can be applied to patients of other races with different body sizes. Thus, the generalizability of the study findings may be limited. Second, the study power may have been limited, because the sample size was not calculated beforehand. Third, there was no written criterion for extubation, and the decision to extubate as well as the use of steroids before extubation was dependent on the doctors in-charge. Fourth, measurement errors could occur when measuring the length of the airway size in CT images. In addition, the size of the trachea changes depending on the timing of breathing. In particular, the vocal cords can open and close. Fifth, we did not include patient severity and fluid balance which might affect the length of intubation or systemic edema and subsequently cause post-extubation upper airway obstruction. In particular, we did not evaluate the Simplified Acute Physiology Score (SAPS II), which is associated with post-extubation stridor [22]. When we collected this information, it might have affected the results in either direction. However, there is no single severity score that represents the severity of various patients in mixed ICUs. Thus, our findings regarding tube size might be applicable to patients with different backgrounds. Sixth, the same researchers conducted research planning and statistical analysis, which might have introduced a potential bias and overestimated the results of CT image measurement. Finally, this was an observational study; therefore, causation could not be implied.

The results of this study indicate that assessment of CT scan, which is a popular examination in the emergency department, could be a candidate for the detection of post-extubation upper airway obstruction in female patients. Furthermore, conducting a multicenter, prospective, interventional study might be needed to verify our findings.

## Conclusions

In female patients, no gap between endotracheal tube and the vocal codes or the glottic transverse diameter is smaller than the endotracheal tube outer diameter in CT scan was associated with post-extubation upper airway obstruction symptoms. This result may aid in choosing the appropriate endotracheal tube size in females as well as facilitate early detection of patients at high risk of developing post-extubation upper airway obstruction. Further studies are required to verify these findings.

## Data Availability

The datasets used and/or analyzed during the current study are available from the corresponding author on reasonable request.

## Abbreviations

CT: Computed tomography
ICU: Intensive care unit
OD: Outer diameter
IQR: Inter-quartile range
OR: Odds ratio
CI: Confidence interval
